# How much time do emergency department physicians spend on medication-related tasks? A time- and-motion study

**DOI:** 10.1186/s12873-024-00974-3

**Published:** 2024-04-09

**Authors:** Tine Johnsgård, Renate Elenjord, Renata Vesela Holis, Marit Waaseth, Birgitte Zahl-Holmstad, Marie Fagerli, Kristian Svendsen, Elin Christina Lehnbom, Eirik Hugaas Ofstad, Torsten Risør, Beate Hennie Garcia

**Affiliations:** 1 Hospital Pharmacy of North Norway Trust, Tromsø, Norway; 2 Department of Pharmacy, Faculty of Health Sciences, UiT the Arctic University of Norway, Tromsø, Norway; 3 Department of Community Medicine, Faculty of Health Sciences, UiT the Arctic University of Norway, Tromsø, Norway; 4 Department of Medicine, Nordland Hospital Trust, Bodø, Norway; 5 Department of Public Health, Faculty of Health and Medical Sciences, University of Copenhagen, Copenhagen, Denmark

**Keywords:** Emergency department, Physicians, Medication reconciliation, Workflow, Time, Medication errors, Observations

## Abstract

**Background:**

Medication-related problems are an important cause of emergency department (ED) visits, and medication errors are reported in up to 60% of ED patients. Procedures such as medication reconciliation and medication review can identify and prevent medication-related problems and medication errors. However, this work is often time-consuming. In EDs without pharmacists, medication reconciliation is the physician’s responsibility, in addition to the primary assignments of examining and diagnosing the patient. The aim of this study was to identify how much time ED physicians spend on medication-related tasks when no pharmacists are present in the EDs.

**Methods:**

An observational time-and-motion study of physicians in three EDs in Northern Norway was conducted using Work Observation Method by Activity Timing (WOMBAT) to collect and time-stamp data. Observations were conducted in predefined two-hour observation sessions with a 1:1 relationship between observer and participant, during Monday to Friday between 8 am and 8 pm, from November 2020 to October 2021.

**Results:**

In total, 386 h of observations were collected during 225 observation sessions. A total of 8.7% of the physicians’ work time was spent on medication-related tasks, of which most time was spent on oral communication about medications with other physicians (3.0%) and medication-related documentation (3.2%). Physicians spent 2.2 min per hour on medication reconciliation tasks, which includes retrieving medication-related information directly from the patient, reading/retrieving written medication-related information, and medication-related documentation. Physicians spent 85.6% of the observed time on non-medication-related clinical or administrative tasks, and the remaining time was spent standby or moving between tasks.

**Conclusion:**

In three Norwegian EDs, physicians spent 8.7% of their work time on medication-related tasks, and 85.6% on other clinical or administrative tasks. Physicians spent 2.2 min per hour on tasks related to medication reconciliation. We worry that patient safety related tasks in the EDs receive little attention. Allocating dedicated resources like pharmacists to contribute with medication-related tasks could benefit both physicians and patients.

**Supplementary Information:**

The online version contains supplementary material available at 10.1186/s12873-024-00974-3.

## Introduction

Emergency departments (EDs) are high-paced work environments where different healthcare professionals (HCPs) work together to provide care for patients with various medical issues. Physicians play a key role in the EDs, often multitasking under time pressure. In addition to having the main responsibility for patient assessment and diagnosing, physicians initiate appropriate therapy and make decisions regarding admission or discharge. A central part of this process includes obtaining and documenting the patient’s medical history and medication list [1, 2]. Correct information is essential for making appropriate decisions regarding treatment during admission, and to prevent medication discrepancies during transitions of care. Medication reconciliation (MedRec) is a process ensuring correct information about patients’ medication use, and while HCPs recognize the value of MedRec, a lack of agreement regarding HCPs’ roles and responsibilities in the process has been identified [3]. In some countries, pharmacists have been introduced as a part of the ED interprofessional team, having the responsibility for some of the medication-related work tasks that in other countries are the responsibility of physicians, e.g., MedRec [4, 5].

Medication-related problems are an important cause of ED visits [6–8]. Medication errors are reported in up to 60% of ED patients [9, 10], and can lead to hospitalizations and even deaths [11]. Early identification of medication errors and medication-related problems through tasks like MedRec and medication review can prevent hospitalization, reduce length of stay and improve therapy [12, 13]. However, this work demands time and attention, and studies have shown that these tasks have low priority among ED physicians [14, 15]. In Norway, MedRec is the physicians’ responsibility and in a recent publication, Norwegian ED physicians describe MedRec as time-consuming detective work where they often have to make decisions based on contradictory information from several different sources [16].

Previous studies have investigated how physicians in EDs spend their time [17–19]. However, as the applied task categories vary between studies, it is hard to compare their results. To our knowledge, only one study has specifically focused on the proportion of time spent on medication-related tasks [4]. This Norwegian study from 2022 found that physicians spent about 18% of their time on medication-related tasks in an ED where the clinical pharmacist was present [4]. The most time-consuming medication-related task was to gather information about medication use (part of MedRec), of which physicians spent 7% of the observed time.

The aim of the present study was to identify how much time ED physicians spend on medication-related tasks with no pharmacists present. We also investigated how much time ED physicians spend on the MedRec process.

## Methods

### Study design and setting

This was an observational time-and-motion study of physicians in three EDs in Northern Norway, applying the validated Work Observation Method by Activity Timing (WOMBAT) methodology which allows for collection of time-stamped observational data [20, 21]. The study was designed and reported according to the “Suggested Time And Motion Procedures (STAMP)” guidelines [22] and STROBE statement [23]. Observations were performed between November 13, 2020, to October 15, 2021.

We observed physicians in EDs located in three urban specialist healthcare hospitals. The annual admission rates were approximately 6000 (ED1), 13.000 (ED2) and 16.000 (ED3) patients. ED1 has mainly junior physicians (1–2 years of experience) present in the ED, and senior physicians (≥ 3 years of experience) on call in the hospital. ED2 and ED3 have both junior and senior physicians present in the ED. ED2 was the only hospital with emergency medicine specialists present in the ED to supervise and help junior and senior physicians (weekdays 8 a.m. to 4 p.m.). ED3 is a part of a university hospital and provides specialized services for patients from the northern part of Norway, including the areas covered by the hospitals housing ED1 and ED2.

In Norway, patients arriving at the EDs are usually referrals from primary care (e.g., general practitioner or municipal emergency clinic) or transfers from other hospitals. Severely ill patients or patients with acute trauma can also arrive directly by ambulance. Most often, patients are first seen by an ED nurse who uses the Rapid Emergency Triage and Treatment System (RETTS) to determine the urgency of the situation [24, 25]. Depending on severity, a junior or senior physician examines the patient. Physicians from different departments provide care for their respective patients. Most often, junior physicians take a medical history including a medication history, perform MedRec and compile a medication list. In Norway, medication lists are not automatically shared between different care settings, and there are many sources to consult for information when performing MedRec. In addition to talking to patients, next-of-kin, and nursing homes etc., physicians can access and read from other sources (Table 1). After taking a medical history, the physicians further decide on a treatment plan and determine whether admission is necessary.

**Table 1:** (Norwegian) Sources for reading and retrieving information about medication use during medication reconciliation

Sources	Contains
Summary Care Record	A selection of key health data and complete overview of prescribed and dispensed medications with 3 years medication history. Access: all healthcare professionals
Prescription Intermediary	Database with all valid electronic prescriptions. Strength: prescription information can be imported to the medication module in the electronic health record. Limitations: only 30 days medication history and paper prescriptions are not shown. Access: only prescribers
Medication module in the electronic health record	The hospitals electronic documentation of a patient’s medications. If not reconciled and updated with prescriptions from the Prescription Intermediary upon admission, old prescriptions from previous hospitalizations can become part of the medication list. A table with the medication list can be automatically inserted in patient records. This list forms the basis for medication information throughout the hospital stay
Medication chart	Paper list with the patient’s current medications, used for documentation of prescribed and administered medications during hospitalization. The medication chart at ED1 and ED3 was a printed version from the medication module in the electronic health record. For ED2 the medication chart was handwritten

### Sample size and recruitment

**Fig. 1:**
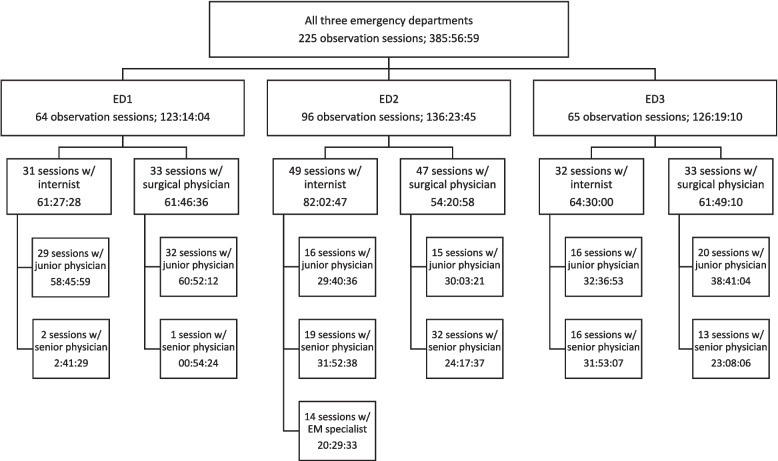
Distribution of observation sessions and total observation time per physician category and emergency department (ED) EM = emergency medicine

We aimed to achieve equal observation time of physicians within the following categories: 1) junior internist, 2) junior surgical physician, 3) senior internist, 4) senior surgical physician and 5) emergency medicine specialist in ED2 (see Fig. 1). We planned for 30 h observation time per physician category in each ED, in total 120–150 h per ED. The number of observation hours were based on previous studies using WOMBAT and determined as sufficient for the purpose of the study [26–28].

Physicians were informed about the study through e-mails, Facebook groups and department meetings. We recruited physicians daily during the observation period, by showing up in the ED asking them directly to participate. All ED physicians at work during the observation period were eligible for inclusion. The internists and surgeons are affiliated with different hospital wards, with roster-based shifts in the ED, leading to frequent changes of on-duty physicians in the ED. We strived to observe different physicians each time. All but three approached physicians agreed to be observed.

### Task categories and piloting

We developed the task categories for the WOMBAT software based on open observations in the EDs (TJ, RVH). Inspiration for categories and definitions was also gathered from a similar Norwegian study [4], and an Australian study [29]. Five observation dimensions with categories and subcategories were developed describing 1) *What* task was done (Table 2), 2) *Where* the task took place, 3) *Who,* if anyone, the task was done with, 4) *How* the task was done, and 5) a consecutive unique number of the *patient* treated, communicated with or about during the observation session.

**Table 2:** The categories and subcategories under the dimension “WHAT”

Categories	Subcategories
Patient examination/treatment	-
Oral communication	Retrieve medication-related information
	Give medication-related information
	Communication about medications
	Work-/patient-related
Read/retrieve written information	-
Documentation	Medication-related
	Non-medication-related
Movement	-
Medication management	Medication preparation without patient
	Preparation and administration of medications with patient
	Double checking
Waiting/consideration	-
Logistics	Medication-related
	Other
Standby	-
Meeting	-
Unknown	-
Other	-

The WOMBAT dimensions and categories were piloted in all three EDs by several members of the research team (TJ, RVH, MF, ECL), and adjusted accordingly. Observers (MF, RVH) received training and supervision by an experienced researcher (ECL). A total of 50 h of training and piloting were conducted by the two observers prior to data collection. See Supplementary 1 for the final version comprising five dimensions, 32 categories and 25 subcategories, and Supplementary 2 for definitions and examples of the “what” categories and subcategories. All tasks were defined as either medication-related, non-medication-related clinical or administrative, or other. The “read/retrieve written information” category was defined as medication-related if this was apparent from the how-dimension (i.e., if the Summary Care Record, Prescription Intermediary, Chart, or medication module in electronic health record were used). We defined the MedRec process to include the following medication-related tasks in the *What*-dimension; 1) oral communication – retrieve medication-related information, 2) read/retrieve written information, and 3) documentation – medication related.

### Data collection and validation

Two observers (MF, Master of Pharmacy student, and RVH, clinical pharmacist/post-doctoral research fellow) collected data in pre-defined two-hour sessions with maximum three sessions per day to minimize observer fatigue. MF collected data in ED1 and ED3, and RVH collected data in ED2. The observation sessions were pre-scheduled to ensure equal distribution of observation time throughout all weekdays, and covered the working hours of ED pharmacists in a planned future intervention study [30]: Monday to Friday between 8 am and 8 pm. Observations were conducted in a 1:1 relationship between observer and participant.

Reliability testing was conducted prior to and during the data collection period to ensure highest possible level of inter-observer agreement. We arranged seven 20 min-sessions where two observers simultaneously observed and registered tasks conducted by the same participant. If the agreement was too low, a new session was conducted until reaching excellent agreement between observers [31, 32].

### Data management and analysis

Data was collected using an iPad® Mini with WOMBAT software version 3.0 installed, which provides quick and easy transition between task categories. Data management and analysis were performed applying Microsoft Excel© (version 2014), IBM SPSS Software© (version 29.0) and SAS Software© (version 9.4).

Data is presented descriptively with total observation time (hours:minutes:seconds), proportions (%) or medians (range). Proportion of time spent on single tasks was calculated as ´total time spent on the task´ (including multitasking), divided by ´total time of observation´. When including multitasking in the proportion calculations, the proportions add up to more than 100%. ‘Active task time’ is total observation time excluding time for being standby or in movement. The 95% confidence intervals (CIs) for proportions of time per task (what categories) were calculated by a bootstrap approach using a SAS Macro program developed for WOMBAT data [33]. Statistically significant differences were defined as non-overlapping 95% CIs.

### Ethics

All participants supplied a signed written consent. Patients were informed that an observer of the physician was present. The study was approved by the Data Protection Officer at Hospital Pharmacy of North Norway Trust (no. 02330).

## Results

A total of 225 observation sessions (Fig. 1) resulted in 386 h of observations (total observation time). Including multitasking, this corresponds to 412 h of data (total time). Median session time was 2.0 h (2 h: 0 min: 18 s).

Physicians spent 85.6% of observed time on non-medication-related clinical or administrative tasks, of which work-/patient-related oral communication (33.3%, 95% CI: 32.2–34.3), reading/retrieving written information (14.4%, 95% CI: 13.5–15.2) and documentation (15.9%, 95% CI: 14.9–17.0) were the most time-consuming tasks. 11.7% (95% CI: 10.6–12.6) was spent on patient examination or treatment (direct patient care). Physicians spent 8.7% of observed time on medication-related tasks, and the most time-consuming tasks were documentation (3.2%, 95% CI: 2.8–3.6) and oral communication about medications (3.0%, 95% CI: 2.8–3.3). The remaining time (12.4%) was spent standby or moving between tasks. Junior physicians spent significantly more time on the three MedRec tasks than senior physicians (Table 3). See Supplementary 3 for results per ED.

**Table 3:** Total observed time and proportion of time for physicians across all task categories

Categories of the “What” dimension	Junior physicians (250:40:05^a^)			Senior physicians (135:16:54^a^)			Total (385:56:59^a)^		
	Time (h:min:s)	%	(95% CI)	Time (h:min:s)	%	(95% CI)	Time (h:min:s)	**%**	(95% CI)
**Non-medication-related clinical or administrative tasks**	213:33:56	85.2	-	116:54:29	86.4	-	330:28:25	85.6	-
Patient examination/treatment	30:02:40	12.0	(10.6–13.3)	14:58:58	11.1	(9.4–12.4)	45:01:38	11.7	(10.6–12.6)
Oral communication	73:08:55	29.2	**(28.1–30.4)**	55:22:50	40.9	**(38.9–42.9)**	128:31:45	33.3	(32.2–34.3)
Read/retrieve written information	32:13:50	12.9	**(11.9–13.9)**	23:12:18	17.2	**(15.6–18.9)**	55:26:08	14.4	(13.5–15.2)
Documentation	49:00:26	19.6	**(18.3–21.1)**	12:11:16	9.0	**(7.8–10.2)**	61:11:42	15.9	(14.9–17.0)
Waiting/consideration	8:48:04	3.5	(3.1–4.1)	4:41:35	3.5	(2.8–4.4)	13:29:39	3.5	(3.1–4.0)
Logistics	3:58:21	1.6	(1.4–1.8)	1:37:13	1.2	(1.0–1.4)	5:35:34	1.4	(1.3–1.6)
Meeting	6:46:15	2.7	(1.6–3.6)	2:19:18	1.7	(0.2–4.1)	9:05:33	2.4	(1.4–3.2)
Unknown	9:16:28	3.7	(2.2–5.0)	2:29:25	1.8	(0.9–3.2)	11:45:53	3.0	(2.0–3.9)
Other	0:18:57	0.1	(0.0–0.3)	0:01:36	0.02	(0.0–0.04)	0:20:33	0.1	(0.0–0.2)
**Medication-related tasks**	23:29:44	9.4	-	9:59:14	7.4	-	33:28:58	8.7	-
Oral communication:	9:39:05	3.9	-	7:37:18	5.6	-	17:16:23	4.5	-
Retrieve medication information^b^	3:48:34	1.5	**(1.3–1.8)**	0:56:34	0.7	**(0.5–0.8)**	4:45:08	1.2	(1.1–1.4)
Give medication information	0:32:38	0.2	(0.2–0.3)	0:22:50	0.3	(0.2–0.4)	0:55:28	0.2	(0.2–0.3)
About medications	5:17:53	2.1	**(1.9–2.4)**	6:17:54	4.7	**(4.0–5.2)**	11:35:47	3.0	(2.8–3.3)
Read/retrieve written information^b^	2:35:56	1.0	**(0.9–1.3)**	0:45:27	0.6	**(0.3–0.8)**	3:21:23	0.9	(0.7–1.0)
Documentation^b^	10:55:00	4.4	**(3.7–4-8)**	1:32:33	1.1	**(0.8–1.5)**	12:27:33	3.2	(2.8–3.6)
Logistics	0:00:00	0.0	-	0:00:00	0.0	-	0:00:00	0.0	-
Medication management	0:19:43	0.1	(0.0–0.3)	0:03:56	0.05	(0.01–0.1)	0:23:39	0.1	(0.0–0.2)
**Other**	28:53:01	11.5	-	19:08:18	14.1	-	48:01:19	12.4	-
Movement	8:37:49	3.4	**(3.2–4.0)**	6:20:43	4.7	**(4.2–5.1)**	14:58:32	3.9	(3.7–4.3)
Standby	20:15:12	8.1	(6.6–9.5)	12:47:35	9.5	(7.5–11.4)	33:02:47	8.6	(7.4–9.7)

Bold numbers show non-overlapping 95% CIs between junior and senior physicians’ time distribution

^a^Proportions were calculated using total observation time (hours:minutes:seconds) as denominator. The proportions add up to more than 100% due to multitasking

^b^The three medication reconciliation tasks

### Medication-related tasks

Of the observed time spent on medication-related tasks (Table 4), 34.6% was spent on communication about medications with other HCPs, 16.8% on documenting medications on charts, 14.2% on orally retrieving information about patients’ medication use, 12.0% on documenting medications in the medication module in the electronic health record, and 10.0% on retrieving written information about patients’ medication use.

**Table 4:** Total observed time and proportion of time across medication-related tasks, including “with whom” or “how” the task is performed

What	**With whom/how**	Junior physicians (23:29:44^a^)		Senior physicians (9:59:14^a^)		Total (33:28:58^a^)	
		Time (h:min:s)	%	Time (h:min:s)	%	Time (h:min:s)	%
**Oral communication**		**9:39:05**	**41.1**	**7:37:18**	**76.3**	**17:16:23**	**51.6**
**Retrieve medication information**^b^		**3:48:34**	**16.2**	**0:56:34**	**9.4**	**4:45:08**	**14.2**
	Patient	3:36:27	37.4	0:54:02	9.0	4:30:29	13.5
	Next-of-kin	0:26:44	1.9	0:00:48	0.1	0:27:32	1.4
	Source outside hospital^c^	0:07:04	0.5	0:00:00	0.0	0:07:04	0.4
**Give medication information**		**0:32:38**	**2.3**	**0:22:50**	**3.8**	**0:55:28**	**2.8**
	Patients	0:25:42	1.8	0:18:52	3.1	0:44:34	2.2
	Next-of-kin	0:06:59	0.5	0:04:24	0.7	0:11:23	0.6
**About medications**		**5:17:53**	**22.5**	**6:17:54**	**63.1**	**11:35:47**	**34.6**
	Nurse	1:09:24	4.9	0:49:00	8.2	1:58:24	5.9
	Junior physician	0:53:11	3.8	2:05:13	20.9	2:58:24	8.9
	Senior physician	2:24:32	10.3	1:25:51	14.3	3:50:23	11.5
	Medical student	1:17:37	5.5	0:14:25	2.4	1:32:02	4.6
	Specialist physician	0:05:59	0.4	0:23:39	3.9	0:29:38	1.5
	Patient	0:06:38	0.5	0:21:53	3.7	0:28:31	1.4
	Pharmacists	0:00:00	0.0	0:00:00	0.0	0:00:00	0.0
	Next-of-kin	0:00:00	0.0	0:04:40	0.8	0:04:40	0.2
	Nurse coordinator	0:01:38	0.1	0:04:49	0.8	0:06:27	0.3
	Source inside hospital	0:03:25	0.2	0:44:44	7.5	0:48:09	2.4
	Source outside hospital^c^	0:02:35	0.2	0:21:28	3.6	0:24:03	1.2
	Other	0:00:00	0.0	0:06:58	1.2	0:06:58	0.3
**Read/retrieve written information**^b^		**2:35:56**	**11.1**	**0:45:27**	**7.6**	**3:21:23**	**10.0**
	Summary Care Record	1:30:59	6.5	0:29:20	4.9	2:00:19	6.0
	Prescription intermediary	0:39:43	2.8	0:11:41	1.9	0:51:24	2.6
	Medication module in electronic health record	1:16:46	5.4	0:19:06	3.2	1:35:52	4.8
	Medication chart	0:09:07	0.6	0:03:26	0.6	0:12:33	0.6
**Documentation**^b^		**10:55:00**	**46.5**	**1:32:33**	**15.4**	**12:27:33**	**37.2**
	Medication chart	4:59:43	21.3	0:37:07	6.2	5:36:50	16.8
	Medication module in electronic health record	3:48:34	16.2	0:11:47	2.0	4:00:21	12.0
	Other	2:06:43	9.0	0:43:39	7.3	2:50:22	8.5

^a^Proportions are calculated using the observed medication-related time (hours:minutes:seconds) as denominator

^b^The three medication reconciliation tasks

^c^Sources outside hospital, e.g., pharmacies, nursing homes and home care nurses

Bold font show the "what" categories and subcategories

The patient was the most frequently used source of orally retrieved information about his/her medication use (13.5%). Only junior physicians contacted other information sources like nursing homes, home care nurses or pharmacies. When physicians communicated about medications, they most often communicated with other physicians (junior and senior), including medical students. When physicians were reading or retrieving written information about patients’ medication use, they spent most time retrieving information from the Summary Care Record and from the medication module in the electronic health record.

MedRec tasks were observed in 177 of the 225 observation sessions and for 298 unique patients. Physicians spent 6.1% of their active task time on these tasks, corresponding to median 2.2 min per hour (minimum 5.5 s and maximum 16.4 min). Junior internists spent 10.0% of their active task time on MedRec (median 5.3 min per hour), while junior surgical physicians spent 5.7% of their active task time on MedRec (median 2.5 min per hour). See Table 5.

**Table 5:** Time and proportion of time spent on medication reconciliation (MedRec) tasks

**Table**	**Junior internists** Time (h:min:s)	**Junior surgical physicians** Time (h:min:s)	**All physicians** Time (h:min:s)
Active task time^a^	109:20:40	112:26:24	337:55:40
Observed time^b^	10:57:58	06:21:32	20:34:04
% time^a^	10.0	5.7	6.1
Median time/session	00:10:41	00:05:05	00:04:18
Min. time/session	00:02:17	00:00:17	00:00:11
Max. time/session	00:32:05	00:23:17	00:32:43
No. sessions w/MedRec	56	57	177
No. patients in sessions w/MedRec	110	98	298

^a^Active task time is total observation time excluding time for movement and standby and is used as denominator when calculating proportions in each group

^b^Observed active task time spent on MedRec tasks: 1; oral communication – retrieve medication-related information, 2; read/retrieve written information; from Summary Care Record, Prescription Intermediary, medication module in electronic heath record and chart, and 3; medication-related documentation

## Discussion

This is, to our knowledge, the first study that specifically focus on the time physicians spend on medication-related tasks in EDs without pharmacists present. We observed that 8.7% of physicians’ time was spent on medication-related tasks, while the majority (85.6%) of time was spent on non-medication-related clinical or administrative tasks. This study describes the baseline of work time distribution for ED physicians before the implementation of clinical pharmacists in the interprofessional team [30]. Future studies can thereby investigate how pharmacists impact physicians’ use of time in the same EDs.

Medication-related problems are important causes of ED visits [6–8] and medication errors occur frequently in ED patients [9, 10]. Adding the consequences related to readmissions, morbidity and mortality [6, 34, 35], it is surprising that physicians in our study spent less than 10% of their time on medication-related tasks. A previous Norwegian study from 2022 found that physicians in EDs spent 17.8% of their time conducting medication-related tasks [4]. The EDs in our study and the previous Norwegian study are similar with regards to observation times, admission rates and physicians working shifts in the EDs, but one important difference was the part-time presence of the ED pharmacist in the study by Nymoen et al. On the one hand, one could assume that the time physicians spend on medication-related tasks would be *lower* with the presence of pharmacists taking responsibility for time-consuming activities like for instance MedRec. On the other hand, the presence of ED pharmacists acts as a reminder to physicians to be more aware of and prioritize medication-related tasks and thus spend *more* time on them. This spill-over effect can be explained by the theory of “three degrees of influence”, saying that our (e.g., pharmacists’) words and actions influence others (e.g., physicians), and most influence is seen for those with whom we are directly connected [36]. Professional communication between physicians and pharmacists in Nymoen’s study accounted for only 0.04% of the medication-related tasks performed by physicians, so the interaction itself cannot explain why physicians in Nymoen’s study spent twice as much time on medication-related tasks compared with our study. Nonetheless, spending more time on medication-related tasks could be seen as a means to increase patient safety. Future studies should investigate optimal use of time and effort for patient safety tasks, and which tasks each HCP should contribute with.

With regards to MedRec, we were surprised by finding that only 2.2 min per hour was dedicated to this task, given the importance of identifying the correct use of medications at admission [37]. In comparison, this is 5.5 min less than the study by Nymoen *et.al* who reported 7.8 min per hour on MedRec tasks [4]. The observed time for MedRec is interestingly much shorter than what physicians convey in a previous study [16]. In a qualitative interview study of 27 physicians from the same three EDs, physicians perceived MedRec as ‘a very time-consuming task’ demanding high effort [16]. In addition, they perceived the MedRec process as overwhelming and burdened with uncertainty. This, as well as the general state of time pressure in the ED, was a reason why physicians in the same study expressed that they wanted work relief by ED pharmacists [16]. The gap between actual time and perceived time in these two studies indicates that physicians associate a high cognitive burden with the MedRec process, which should be taken into consideration when allocating resources to different work tasks in the ED. Our results also show a tendency for surgical physicians to spend less time on MedRec than internists. Potential reasons for this may include surgical patients having fewer medications or that internists focus more on medications compared to surgical physicians. It could also be that internists and surgical physicians are trained differently and have different workflows. Future studies are needed to shed light on this.

With extensive knowledge about medications and medication use, ED pharmacists play an important role in the ED team in other countries [38–40]. In a Spanish study from 2017, 57.2% of the medication errors detected and intervened on by pharmacists were considered severe, and the authors suggested that emergency care would benefit from services provided by pharmacists [39]. Norwegian studies show that up to 80% of hospital medication lists contain medication discrepancies [37, 41], it is therefore necessary to increase focus on MedRec at transitions and ensure correct medication lists in hospitals. By allocating ED pharmacist resources to perform MedRec, physicians’ work burden is relieved, and may allow for increased cognitive capacity to concentrate on other essential work tasks in the ED [15]. In addition, pharmacists can contribute to early identification of medication-related problems and medication errors through tasks like medication review and patient counselling [13, 42]. We argue that placing pharmacists in the ED team represents a great potential to improve patient safety tasks in the ED, as physicians’ work burden is relieved, and patients’ medicines are reviewed by both physicians and pharmacists.

### Strengths and limitations

The main strength of this study is the observation methodology with predefined task categories, observation schedules and time-stamped WOMBAT-data, reducing observation bias and observation fatigue and ensuring accuracy of calculated time [26]. Other strengths include that data were collected from three EDs over the course of a year, we observed different physicians, and we ensured inclusion of physicians with different experience and medical specialties. Time spent on medication-related tasks is quite similar in the three EDs, indicating that the results are probably representative to other ED settings in Norway as well. The high number of observed hours from the three EDs are comparable to other studies of work patterns [4, 43] and observations are spread from Monday to Friday between 8 am and 8 pm which provides a realistic, generalizable, representation of ED physicians’ work time distribution during the day. Altogether, these measures ensure a data material representative of the work time distribution for ED physicians in North Norway, across hospitals, physician specialty and variation over time in admission rate and staffing.

The main limitation of this study is that we have not followed single patients and are not able to calculate how much time is spent on different tasks per patient. Other limitations include that our results are not representative for nights and weekends, as observations were not performed at these times. Rather, our results represent physicians’ work time distribution during the busiest workhours of the three EDs. Another limitation to our study is that senior surgical physicians in ED2 were not always present in the ED during the entire observation session, leading to a few sessions being cut shorter than two hours. We had a similar challenge in ED1, where senior physicians rarely were present in the ED. Consequently, we were forced to change observation strategy by increasing the number of observation sessions of junior physicians instead. Also, using two different observers could introduce inter-observer bias, however we took steps to ensure a high inter-rater agreement between observers through tests of agreement both before and during the data collection period.

## Conclusion

This study shows that physicians working in EDs without pharmacists employed, spend 8.7% of their work time on medication-related tasks. The two most time-consuming medication-related tasks concern oral communication about medications with other HCPs and medication-related documentation. Medication reconciliation accounts for 2.2 min per hour. Results from this study indicate that medication safety tasks in the ED receive little attention. Allocating dedicated resources like pharmacists to contribute with medication-related tasks in the ED could be beneficial for both physicians and patients. In addition to conducting studies investigating patient outcomes, future research should investigate whether physicians’ perception of time and actual time spent on medication-related tasks changes when introducing the ED pharmacist.

### Supplementary Information

**Supplementary Material 1.**

**Supplementary Material 2.**

**Supplementary Material 3.**

## Data Availability

The dataset used and analyzed during the current study are available from the corresponding author on reasonable request.

## Abbreviations

ED: Emergency Department
HCP: Healthcare professional
MedRec: Medication Reconciliation
WOMBAT: Work Observation Method by Activity Timing
STAMP: Suggested Time And Motion Procedures
RETTS: Rapid Emergency Triage and Treatment System
