# Supplementary material for: How much time do emergency department physicians spend on medication-related tasks? A time- and-motion study
Source: BMC Emerg Med. 2024 Apr 9;24:56. doi: 10.1186/s12873-024-00974-3 (PMC11003058; doi:10.1186/s12873-024-00974-3)
Supplement: Supplementary file 1 — Supplementary Material 1. [file 12873_2024_974_MOESM1_ESM.docx]

# Supplementary 1

The final version of the WOMBAT outline consisted of five dimensions as illustrated in Figure 1 (in beige). Data was collected using iPad® Mini’s with WOMBAT software version 3.0 installed (see Picture 1 for screenshot). The WOMBAT software allows for collection of timestamped observational data, recording the exact time from when you start/press the current “what” task until a new task is started or interrupted. It provides with quick and easy transition between task categories. *Indicates mandatory dimensions. Current running task is automatically marked in green, and on the left panel the previously time-stamped tasks and current running task are shown.

| 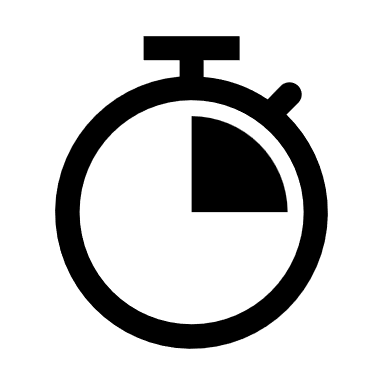Tasks (T) | | WHAT* | | | |
| --- | --- | --- | --- | --- | --- |
| ●Oral com..  T3 19:15:49 | | Patient examination/treatment | Oral communication ↓ | Read/retrieve written information | Documentation ↓ |
| 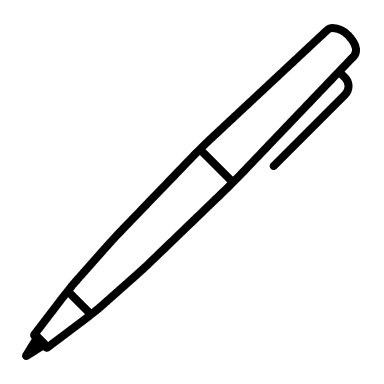Movement  T2 19:15:40 | | Movement | Medication management ↓ | Waiting/consideration | Logistics ↓ |
| **V** Patient examin..  T1 19:15:27 | | Standby | Meeting | Unknown | Other |
|  | | WHERE* | | | |
|  |  | ED | Medication room | Covid19 area | Outside ED |
|  |  | WHO | | | |
|  |  | Patient | Junior physician | Senior physician | Nurse |
|  |  | Nurse coordinator | Unknown | Secretary | Others ↓ |
| 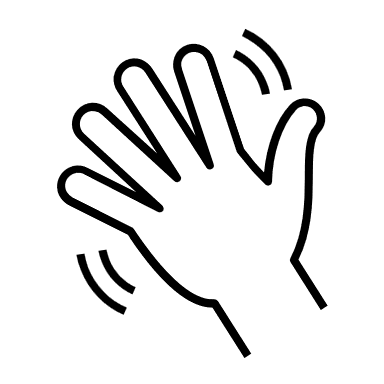 Interrupted | | HOW | | | |
|  | | Face-to-face | Chart | PC ↓ | Telephone |
|  |  | Encyclopedia | Paper journal | Paper | Other |
|  |  | PATIENT | | | |
|  |  | 1 | 2 | 3 | More ↓ |
|  |  | FREETEXT | | | |
|  |  |  | | | |
|  | 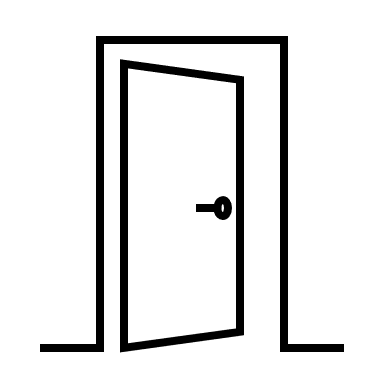 | Next task  Multitask/interrupt | | | |

*Figure 1: Illustration of the WOMBAT software with five dimensions and associated categories.*

1. **WHAT**

Describes which task that is done by the observed physician. This dimension was mandatory. Definitions and examples of categories and subcategories in this dimension is provided in Supplementary 2. Down arrow indicates that there are subcategories chosen from a drop-down menu, consisting of the following:

Oral communication ↓

- Retrieve medication-related information
- Give medication-related information
- Communication about medications
- Work-/patient-related

Documentation ↓

- Medication-related
- Non-medication-related

Medication management ↓

- Medication preparation without patient
- Preparation and administration of medications with patient
- Double checking

Logistics ↓

- Other
- Medication-related

1. **WHERE**

Describes where the observed physician was when conducting the recorded task. This dimension was mandatory, with only one option possible to register.

1. **WHO**

Describes with whom (if anyone) the observed physician performed the task with. This dimension was not mandatory, as many work tasks is conducted without interaction with others. Multiple options possible are to register, e.g., oral communication with a nurse and a senior physician. Down arrow indicates that there are other choices available from a drop-down menu:

Others ↓

- Specialist physician
- Healthcare personnel
- Medical student
- Pharmacist
- Next-of-kin
- Outside hospital

1. **HOW**

Describes practically how the observed physician conducted the task. This dimension was not mandatory, as some tasks don’t require explaining how they are performed, e.g., movement. Multiple options were possible to register, e.g., reading and retrieving information from the prescription intermediary and the Summary Care Record at the same time. Down arrow indicates that there are other choices available from a drop-down menu:

PC ↓

- Electronic Health Record
- Medication module in Electronic Health Record
- Prescription intermediary
- Summary Care Record
- Interaction information screen
- Electronic chart
- Other on PC
- Voice recorder

1. **PATIENT**

Every patient that was treated, communicated with or about during the day of the observation was registered with a unique number. The drop-down menu consisted of numbers up to 30, including “x” which was used if the patient was unknown to the observer. This dimension was not mandatory as not all conducted tasks involves patients, e.g., being standby. Only one option was possible to register.

More ↓

- 4
- 5
- … up to 30
- X (unknown patient)

*Picture 1: Screenshot of the actual WOMBAT software used in the study (with Norwegian language).*
