# Supplementary material for: How much time do emergency department physicians spend on medication-related tasks? A time- and-motion study
Source: BMC Emerg Med. 2024 Apr 9;24:56. doi: 10.1186/s12873-024-00974-3 (PMC11003058; doi:10.1186/s12873-024-00974-3)
Supplement: Supplementary file 2 — Supplementary Material 2. [file 12873_2024_974_MOESM2_ESM.docx]

# Supplementary file 2

“What” dimension’s categories, subcategories, definitions, and examples.

| WHAT CATEGORY | WHAT SUBCATEGORY | DEFINITION | EXAMPLE |
| --- | --- | --- | --- |
| Patient examination/treatment | - | Examination or treatment of the patient (directly) | Measures blood pressure |
| Oral communication | - Retrieve medication-related information | Retrieving information about a patient’s medication use | Talks to e.g., the patient or home care nurse about a patient’s home medications |
|  | - Give medication-related information | Give information that is medication-related | Tells a patient about side effects of morphine |
|  | - Communication about medications | Communication about medications between healthcare personnel | Two physicians discuss a patient’s medication list |
|  | - Work-/patient-related | Communication with or about the patient with healthcare personnel. Work-related communication between colleagues. Not medication-related communication. | Nurse informs physician about triage results |
| Read/retrieve written information | - | Reading in Electronic Health Record or encyclopedia | Read previous discharge notes in Electronic Health Record or checking blood test results |
| Documentation | - Medication-related | Documenting a patient’s medications | Writing medical chart, prescribing medications |
|  | - Non-medication-related | Documenting patient history or blood tests | Writes about a patient’s previous medical history in Electronic Health Record |
| Movement | - | Movement from one place to another, within the ED or between departments | Moving from the break room to the patient room |
| Medication management | - Medication preparation without patient | Preparing medications for the patient | A nurse prepares antibiotic infusion in the medicine room |
|  | - Preparation and administration of medications with patient | Administration of the prepared medication to the patient | A nurse gives a patient the infusion with antibiotic |
|  | - Double checking | Checking (by you or by a colleague) that a medication prepared for administration is done after protocol/prescription | A nurse double checks another nurse to see if he/she prepared the correct medication |
| Waiting/consideration | - | Physician/nurse is not directly active in the work task, could be e.g., thinking, considering or waiting for test results | A nurse waits for results of the urine sample |
| Logistics | - Other | Preparing for the next patient, organizing the day with patients and healthcare personnel. | A nurse cleans a patient room |
|  | - Medication-related | Order medications from hospital pharmacy or other tasks related to medications stated in hospital protocols | Checking the expiration date of the medications available in the medicine room |
| Standby | - | Time spent not doing any specific work tasks. | Lunch/toilet break. Inactive/available time (e.g., no patients in the ED). |
| Meeting | - | Staff meeting, morning meeting, internal teaching/education | Morning meeting where they summarize the previous 24 hours |
| Unknown | - | Not observable work tasks | Due to prevention of infectious diseases the observers can’t join the nurse/physician |
| Other | - | Work tasks inapplicable with the other categories. | A nurse washing hands, completely independent on some of the other work tasks |
