# Supplementary material for: How much time do emergency department physicians spend on medication-related tasks? A time- and-motion study
Source: BMC Emerg Med. 2024 Apr 9;24:56. doi: 10.1186/s12873-024-00974-3 (PMC11003058; doi:10.1186/s12873-024-00974-3)
Supplement: Supplementary file 3 — Supplementary Material 3. [file 12873_2024_974_MOESM3_ESM.docx]

Table S1 Proportion of physicians' work time across all task categories.

| CATEGORIES OF THE “WHAT” DIMENSION | | ED1  123:14:04*  % | ED2  136:23:45*  % | ED3  126:19:10*  % | **Total**  **385:56:59**  **%** |
| --- | --- | --- | --- | --- | --- |
| **NON-MEDICATION-RELATED** **CLINICAL OR ADMINISTRATIVE TASKS** | | **84.6** | **88.8** | **82.9** | **85.6** |
| Patient examination/treatment | | 10.7 | 18.4 | 5.3 | **11.7** |
| Oral communication | | 33.7 | 34.9 | 31.2 | **33.3** |
| Read/retrieve written information | | 9.7 | 15.5 | 17.6 | **14.4** |
| Documentation | | 17.4 | 13.2 | 17.3 | **15.9** |
| Waiting/consideration | | 3.3 | 3.7 | 3.6 | **3.5** |
| Logistics | | 1.9 | 1.0 | 1.6 | **1.4** |
| Meeting | | 4.0 | 0.7 | 2.5 | **2.4** |
| Unknown | | 3.9 | 1.6 | 3.7 | **3.0** |
| Other | | 0.1 | 0.0 | 0.2 | **0.1** |
| **MEDICATION-RELATED TASKS** | | **8.4** | **9.6** | **8.0** | **8.7** |
| Oral communication: | | 3.7 | 5.4 | 4.2 | **4.5** |
|  | Retrieve medication information | 1.4 | 1.4 | 0.9 | **1.2** |
|  | Give medication information | 0.2 | 0.3 | 0.2 | **0.2** |
|  | About medications | 2.2 | 3.7 | 3.0 | **3.0** |
| Read/retrieve written information | | 0.8 | 0.8 | 0.9 | **0.9** |
| Documentation | | 3.6 | 3.3 | 2.8 | **3.2** |
| Logistics | | 0.0 | 0.0 | 0.0 | **0.0** |
| Medication management | | 0.2 | 0.0 | 0.0 | **0.1** |
| **OTHER** | | **10.5** | **12.0** | **14.8** | **12.4** |
| Movement | | 3.5 | 4.5 | 3.6 | **3.9** |
| Standby | | 7.0 | 7.6 | 11.2 | **8.6** |
| *Proportions were calculated using total observation time (hours:minutes:seconds) as denominator. The proportions add up to more than 100% due to multitasking.  ED = emergency department | | | | | |

Table S2 Proportion of time (%) across medication-related tasks, including “with whom” or “how” the task is performed.

| WHAT | WITH WHOM/HOW | ED1 (10:22:23*) | ED2 (13:04:16*) | ED3 (10:02:19*) | Total (33:28:58*) |
| --- | --- | --- | --- | --- | --- |
|  |  | % | % | % | **%** |
| ORAL COMMUNICATION | | **44.2** | **56.7** | **52.6** | **51.6** |
| Retrieve medication information** | | **16.4** | **14.7** | **11.3** | **14.2** |
|  | Patient | 15.6 | 13.4 | 11.3 | **13.5** |
|  | Next-of-kin | 2.7 | 1.0 | 0.5 | **1.4** |
|  | Source outside hospital*** | 0.0 | 0.0 | 1.2 | **0.4** |
| Give medication information | | **2.0** | **3.2** | **2.8** | **2.7** |
|  | Patients | 1.9 | 2.0 | 2.8 | **2.2** |
|  | Next-of-kin | 0.8 | 0.4 | 0.6 | **0.6** |
| About medications | | **25.9** | **38.8** | **38.2** | **34.6** |
|  | Nurse | 6.5 | 3.9 | 7.9 | **5.9** |
|  | Junior physician | 5.6 | 13.3 | 6.4 | **8.9** |
|  | Senior physician | 8.6 | 9.9 | 16.5 | **11.5** |
|  | Medical student | 8.0 | 2.9 | 3.3 | **4.6** |
|  | Specialist physician | 0.1 | 3.7 | 0.0 | **1.5** |
|  | Patient | 0.5 | 0.6 | 3.5 | **1.4** |
|  | Pharmacists | 0.0 | 0.0 | 0.0 | **0.0** |
|  | Next-of-kin | 0.0 | 0.4 | 0.3 | **0.2** |
|  | Nurse coordinator | 0.2 | 0.3 | 0.4 | **0.3** |
|  | Source inside hospital | 0.3 | 4.9 | 1.0 | **2.3** |
|  | Source outside hospital | 0.3 | 1.1 | 2.2 | **1.2** |
|  | Other | 0.0 | 0.0 | 1.5 | **0.5** |
| READ/RETRIEVE WRITTEN INFORMATION** | | **9.9** | **8.8** | **11.7** | **10.0** |
|  | Summary Care Record | 5.5 | 7.1 | 5.1 | **6.0** |
|  | Prescription intermediary | 0.8 | 3.9 | 2.6 | **2.6** |
|  | Medication module in Electronic Health Record | 4.1 | 4.9 | 6.4 | **5.1** |
|  | Chart | 0.9 | 0.2 | 1.0 | **0.6** |
| DOCUMENTATION** | | **42.9** | **34.2** | **35.3** | **37.2** |
|  | Chart | 19.4 | 17.5 | 13.1 | **16.8** |
|  | Medication module in Electronic Health Record | 13.4 | 11.4 | 11.3 | **12.0** |
|  | Other | 10.1 | 5.3 | 10.9 | **8.5** |
| *Proportions are calculated using the observed medication-related time in each emergency department (ED) as denominator. **The three medication reconciliation tasks. ***Sources outside hospital, e.g., pharmacies, nursing homes, home care nurses | | | | | |

Table S3 Time (hours:minutes:seconds) and proportion of time (%) spent on medication reconciliation (MedRec) tasks.

|  | ED1 | | | ED2 | | | ED3 | | | Total | | | |
| --- | --- | --- | --- | --- | --- | --- | --- | --- | --- | --- | --- | --- | --- |
|  | All physicians | Jr. internists | Jr. surgical physicians | All physicians | Jr. internists | Jr. surgical physicians | All physicians | Jr. internists | Jr. surgical physicians | All physicians | Jr. internists | Jr. surgical physicians |  |
| Active task time* | 110:18:17 | 53:10:28 | 54:04:00 | 119:59:58 | 27:17:43 | 26:29:47 | 107:37:25 | 28:52:29 | 31:52:37 | 337:55:40 | 109:20:40 | 112:26:24 |  |
| Observed time** | 07:10:51 | 04:54:37 | 02:07:11 | 07:32:12 | 03:33:22 | 02:18:35 | 05:51:01 | 02:29:59 | 01:55:46 | 20:34:04 | 10:57:58 | 06:21:32 |  |
| % time* | 6.5 | 9.2 | 3.9 | 6.3 | 13.0 | 8.7 | 5.4 | 8.7 | 6.1 | 6.1 | 10.0 | 5.7 |  |
| Median time/session | 00:04:43 | 00:09:58 | 00:02:46 | 00:03:14 | 00:12:28 | 00:05:56 | 00:04:57 | 00:09:36 | 00:06:34 | 00:04:18 | 00:10:41 | 00:05:05 |  |
| Min. time/session | 00:00:10 | 00:00:24 | 00:00:14 | 00:00:09 | 00:02:09 | 00:00:14 | 00:00:13 | 00:04:17 | 00:00:22 | 00:00:11 | 00:02:17 | 00:00:17 |  |
| Max. time/session | 00:45:00 | 00:45:00 | 00:21:09 | 00:30:21 | 00:28:27 | 00:30:21 | 00:22:47 | 00:22:47 | 00:18:22 | 00:32:43 | 00:32:05 | 00:23:17 |  |
| No. sessions w/MedRec | 56 | 26 | 27 | 63 | 16 | 14 | 58 | 14 | 16 | 177 | 56 | 57 |  |
| No. patients in sessions w/MedRec | 100 | 50 | 47 | 99 | 31 | 24 | 99 | 29 | 27 | 298 | 110 | 98 |  |
| *Active task time is total observation time excluding time for movement and standby and is used as denominator when calculating proportions in each group.  **Observed active task time spent on MedRec tasks: 1; oral communication – retrieve medication-related information, 2; read/retrieve written information; from Summary Care Record, Prescription Intermediary, medication module in electronic heath record and chart, and 3; medication-related documentation.  ED = emergency department | | | | | | | | | | | | | |
